# Identification of Flow-Limiting Coronary Stenosis With PCS: A New Cost-Effective Index Derived From the Product of Corrected TIMI Frame Count and Percent Diameter Stenosis

**DOI:** 10.3389/fcvm.2021.718935

**Published:** 2021-11-04

**Authors:** Xinhao Li, Lijuan Lyu, Wei Yang, Jichen Pan, Mei Dong, Mei Zhang, Pengfei Zhang

**Affiliations:** The Key Laboratory of Cardiovascular Remodeling and Function Research, Chinese Ministry of Education, Chinese National Health Commission and Chinese Academy of Medical Sciences, The State and Shandong Province Joint Key Laboratory of Translational Cardiovascular Medicine, Department of Cardiology, Qilu Hospital, Cheeloo College of Medicine, Shandong University, Shandong, China

**Keywords:** coronary artery disease, fractional flow reserve, percutaneous coronary intervention, corrected TIMI frame count, flow-limiting coronary stenosis

## Abstract

**Background:** Identifying functional coronary stenosis with simple and cost-effective methods during invasive coronary angiography is still challenging. Corrected TIMI frame count (CTFC) is considered to be the frame count velocity of coronary blood flow. We aimed to propose a simple and cost-effective index based on CTFC and percent diameter stenosis (DS) to identify flow-limiting coronary stenosis. For this, a new index was put forward as the product of CTFC and DS (PCS). PCS can be regarded as the loss of coronary blood flow due to diameter stenosis.

**Methods:** DS, CTFC, PCS, and Fractional flow reserve (FFR) of 111 vessels in 84 patients with suspected coronary heart disease were measured. FFR ≤0.80 was defined as flow-limiting. Models involving CTFC, DS, and PCS were developed. Logistic regression was performed to evaluate the values on diagnosing flow-limiting stenosis.

**Results:** Vessels with flow-limiting coronary stenosis exhibited higher CTFC values than those without (28.56 vs. 21.64). The performance including the AUC (0.887), sensitivity (87.8%), and Youden index (0.678) for detecting flow-limiting stenosis was improved by adding the CTFC to the DS, while PCS had the largest positive predictive value (PPV) and diagnostic accuracy (DA) being 72.0 and 82.9%, respectively. For vessels with ≥50% lesions, PCS still had the best DA (80.9%), specificity (85.9%), and PPV (72.9%). At the same stenosis severity level, the AUC, Youden index and, DA of PCS were higher than those of CTFC.

**Conclusions:** PCS is simple and accurate to identify flow-limiting coronary stenosis, especially at vessels with moderate to severe stenosis.

## Introduction

Myocardial ischemia is the major cause of cardiovascular adverse events in coronary artery disease (CAD), and its extent and severity are crucial for risk stratification. Patients with extensive ischemia are more likely to benefit from revascularization, but the revascularization of lesions without sufficient evidence of ischemic are also performed and controversial (1, 2). Increasing evidence shows that anatomical coronary artery stenosis does not necessarily indicate the existence of myocardial ischemia, and the functional severity of coronary artery stenosis is the main culprit of myocardial ischemia (3, 4). Therefore, functional evaluation is particularly important for therapeutic strategy making for patients with suspected CAD considered for revascularization (4).

In clinical practice, patients with suspected CAD need to undergo a variety of non-invasive examinations to evaluate ischemia before invasive coronary angiography (ICA). However, most of these examinations cannot lead to conclusive information by a single modality. The results can sometimes be discrepant (5, 6). Fractional flow reserve (FFR) has been recommended as the index to fall back on for determining functional epicardial coronary stenosis during the interventional procedure (4, 5, 7). However, this application is limited due to its complexity, high cost, and risk of complications. In fact, available evidence shows that FFR measurement is rare in invasive coronary angiography (8–10). Obviously, it is wise to make full use of ICA information to acquire physiological information beyond traditional structural information.

CTFC is an index derived from the ICA, calculated as the number of cine frames required for the dye to reach the standardized distal coronary artery marker for the first time. It is a quantitative index to evaluate coronary blood flow with good reproducibility. Studies have shown that corrected TIMI frame count (CTFC) is a predictor of the clinical outcome after thrombolysis or coronary angioplasty (11–14). It is also regarded as an index for myocardial perfusion and microvascular tension evaluation (15, 16). Nevertheless, there are no studies analyzing the combined effects of CTFC and stenosis severity on functional flow-limiting.

**Figure 1:**
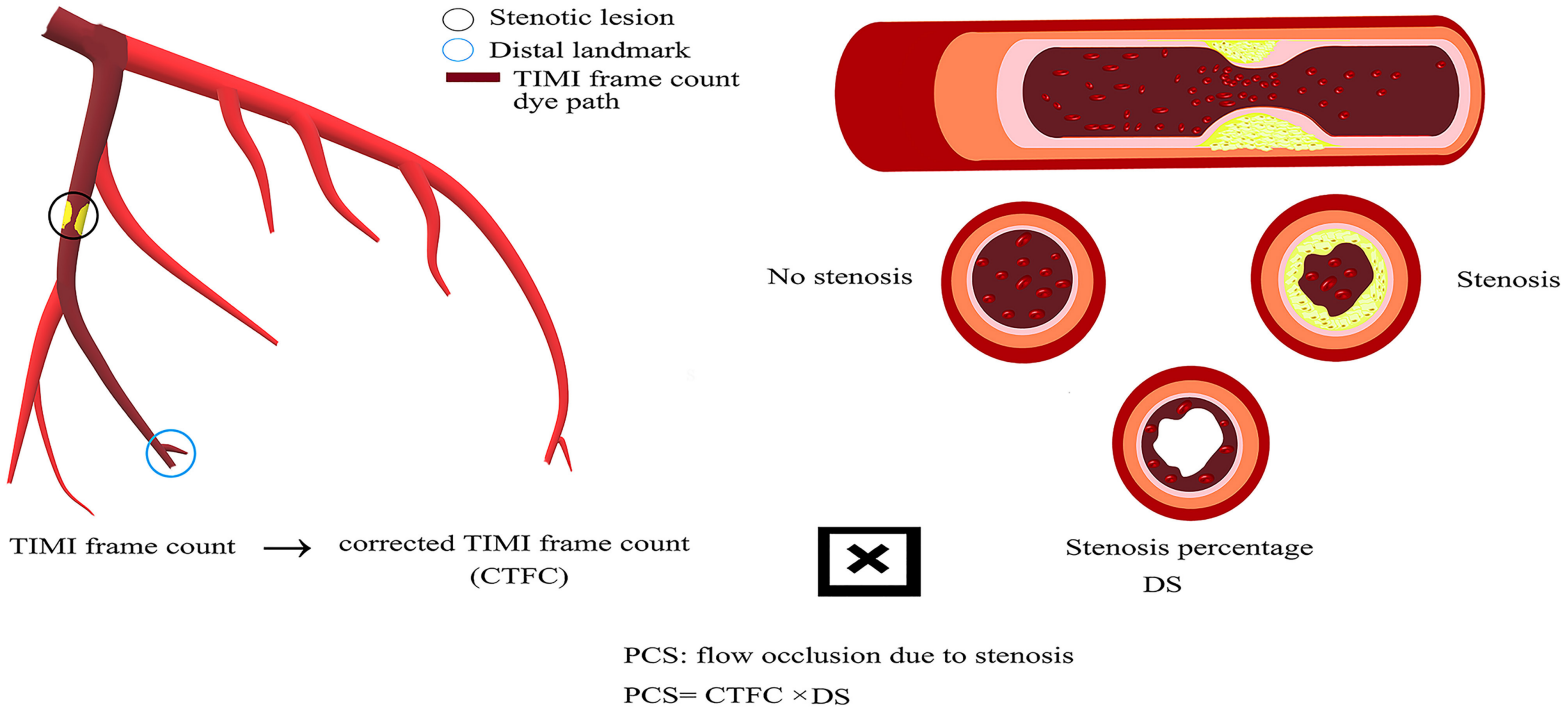
The calculation method of PCS.

We hypothesized that the combination of CTFC and percent diameter stenosis (DS%) from the ICA could provide both physiological and anatomical information. We coincided with a new index called PCS, which is considered as the product of CTFC and DS, to synthesize information. PCS can be regarded as the loss of coronary blood flow due to stenosis (Figure 1). The purpose of this study is to validate the value of PCS in diagnosing flow-limiting coronary stenosis by using FFR as the reference.

## Methods

### Study Design and Participants

This study was the *post hoc* analysis of “Quantitative Analysis of Functional Computed Tomography (CT) Imaging of Coronary Atherosclerosis cohort study” (NCT04986410). From July 1st, 2017 to December 31st, 2020, all the 640 patients (aged 18–85 years old) who presented in our hospital with known or suspected CAD and underwent CT myocardial perfusion, were included in this cohort. Eighty-four from the 640 patients received coronary artery angiography and invasive FFR. Coronary arteries from the 84 patients met the following criteria were included in this study. The inclusion criteria were: (1) at least one lesion with >30% DS; (2) good quality of angiograms. The exclusion criteria were: (1) lesion with >95% DS; (2) feeding collateral vessel; (3) stented coronary artery; (4) bypass graft lesions; (5) thrombotic lesions. This study was approved by the Ethics Committee of Scientific Research of Shandong University Qilu Hospital (KYLL-2016-336) and conducted in accordance with the Declaration of Helsinki.

### ICA Analysis and FFR Measurement

The ICA was performed through radial or femoral artery access. Angiographic images demonstrating the stenosis in its most severe view were used to analyze. DS was analyzed using validated software (Allura 2D-QCA, Phillips Medical System, The Netherlands) by experienced interventional cardiologists double-blindly to FFR and CTFC. DS was quantified as follows: (reference diameter–minimum lumen diameter)/(reference diameter) × 100%. If there were multiple lesions in one coronary artery, we included the most structurally severe lesion in the study. The arteries were subsequently divided into three groups according to DS: mild stenosis (30–49%), intermediate stenosis (50–70%), severe stenosis (>70%). Obstructive coronary artery disease was defined as at least 1 vessel with stenosis ≥50% (17). The operation of FFR was performed in accordance with the standard recommendation (18). Adenosine injection was pumped intravenously at a rate of 160 μg/kg/min for maximal hyperemia (19). FFR ≤ 0.80 was defined as flow-limiting coronary stenosis (4, 19, 20).

Two other interventional cardiologists with more than 5 years of experience reviewed the angiographic data and measured the CTFC blindly to the FFR results. If there was a disagreement, the third author was invited to discuss and come to a final conclusion. CTFC counted the number of frames required for dye to reach standardized distal landmarks at 30 frames/s (21). The first frame was defined as the frame when the dye fully entered the artery. The last frame was defined as the frame at which the dye entered the branch of the distal landmark (21). Considering that the imaging speed of our coronary angiography was 15 frames/s, we multiplied this TIMI frame count by 2. Since the TIMI frame count of the left anterior descending artery (LAD) is ~1.70 times that of the left circumflex artery (LCX) and the right coronary artery (RCA), the longer LAD frame count was corrected by dividing it by 1.70 to obtain the CTFC (21, 22). The product of CTFC and percent diameter stenosis was also calculated as a new index, namely PCS.

### Statistical Analysis

The sample size was calculated according to the top boundary of 0.76 for QCA diagnostic accuracy in the Favor pilot study and 0.80 was selected as the reference target (23). To reach an AUC value of 0.8, with a type I error at alpha = 0.05 and power = 0.8, the minimal sample size needed for this study was 26, calculated by Med Calc® 19.04 software (Med Calc Software BVBA, Ostend, Belgium).

Each variable was checked for normality before statistical analysis. Categorical data are presented as numbers and percentages (%), continuous variables with a normal distribution are presented as the mean (± standard deviation), and non-normally distributed variables are presented as the median (interquartile range). The chi-square (χ^2^) test was used to compare categorical data. For each variable, the Kolmogorov-Smirnov test was used to check the normality. A two-tailed independent group *t*-test was used to analyze normally distributed continuous variables between the two groups, while the Mann-Whitney *U*-test was used to analyze non-normally distributed continuous variables. Pearson or Spearman correlation analysis was used to determine the correlation between study variables. To evaluate the diagnostic value, we performed univariate and multivariate binary logistic regression analysis. Receiver operating characteristic (ROC) curves were then plotted, and areas under the curve (AUCs) were calculated for the variables. The goodness of the logistic regression model fit was tested using Hosmer-Lemeshow goodness-of-fit tests. Thirty vessels were randomly selected to evaluate the reproducibility of CTFC. Coefficient of variation (COV) and Bland-Altman analysis were used to evaluate intra- and interobserver variability. All the results were performed using bilateral 95% confidence intervals (CIs), and two-tailed tests with *p* < 0.05 were considered statistically significant. These statistical analyses were performed using SPSS 25 software (SPSS Inc., Chicago, IL, USA).

## Results

### Baseline Characteristics

A total of 84 patients were analyzed in the study. The mean age was 59.19 ± 11.28 years and 56 patients (66.7%) were male. Thirty-nine patients (46.4%) were current smokers, 20 (23.8%) had diabetes mellitus, 21 (25.0%) had a family history of CAD, 15 (17.8%) had prior myocardial infarction, and 60 (71.4%) had hypertension. Baseline characteristics are shown in Table 1.

**Table 1:** Demographic data and baseline characteristics of vessels.

**Variables**	
**Clinical characteristics**, ***n*** **=** **84**	
Age (years)	59.19 ± 11.28
Male, *n* (%)	56 (66.7)
BMI (kg/*m*^2^)	25.30 ± 2.97
Smoking, *n* (%)	39 (46.4)
Diabetes mellitus, *n* (%)	20 (23.8)
Family history of premature CAD, *n* (%)	21 (25.0)
Prior myocardial infarction *n* (%)	15 (17.8)
Hypertension, *n* (%)	60 (71.4)
HR (beats/min)	71.20 ± 10.92
LVEF (%)	65.36 ± 4.73
T-CHO (mmol/L)	3.66 ± 0.99
TG (mmol/L)	1.37 ± 0.53
HDL-C (mmol/L)	1.06 ± 0.25
LDL-C (mmol/L)	2.08 ± 0.65
**Angiographic characteristics**, ***n*** **=** **111**	
**Lesion location**	
Left main coronary artery	1 (0.9)
Left anterior descending artery	55 (49.5)
Proximal lesion	27
Mid lesion	24
Distal lesion	4
Left circumflex artery	19 (17.1)
Proximal lesion	5
Mid lesion	8
Distal lesion	6
Right coronary artery	36 (32.4)
Proximal lesion	8
Mid lesion	18
Distal lesion	10
Bifurcation lesions	25 (22.5)
Long lesions	45 (40.5)
Simple lesions	50 (45.0)
Percent diameter stenosis (%)	66.48 ± 15.16
Corrected TIMI frame count (frame)	24.20 ± 6.97
PCS (frame)	16.23 ± 6.40
Fractional flow reserve	0.80 ± 0.16

*Data are presented as mean ± SD, or n (%). BMI, body mass index; CAD, coronary artery disease; HR, heart rate; T-CHO, total cholesterol; TG, triglycerides; HDL-C, high-density lipoprotein cholesterol; LDL-C, low-density lipoprotein cholesterol; LVEF, left ventricular ejection fraction*.

### ICA Findings and FFR Results

According to inclusion and exclusion criteria, 111 vessels (1 LMA, 55 LAD, 19 LCX, and 36 RCA) were included and 41 of them (36.9%) were found with positive FFR. Mild stenosis was found in 17 vessels, intermediate stenosis in 50 vessels, and severe stenosis in 44 vessels. Among these, 41 were proximal lesions, 50 were mid lesions, and 20 were distal lesions. Only 26.0% of vessels with intermediate stenosis had positive FFR. Even in coronary arteries with severe stenosis, 38.6% of these lesions did not induce blood flow impairment.

**Figure 2:**
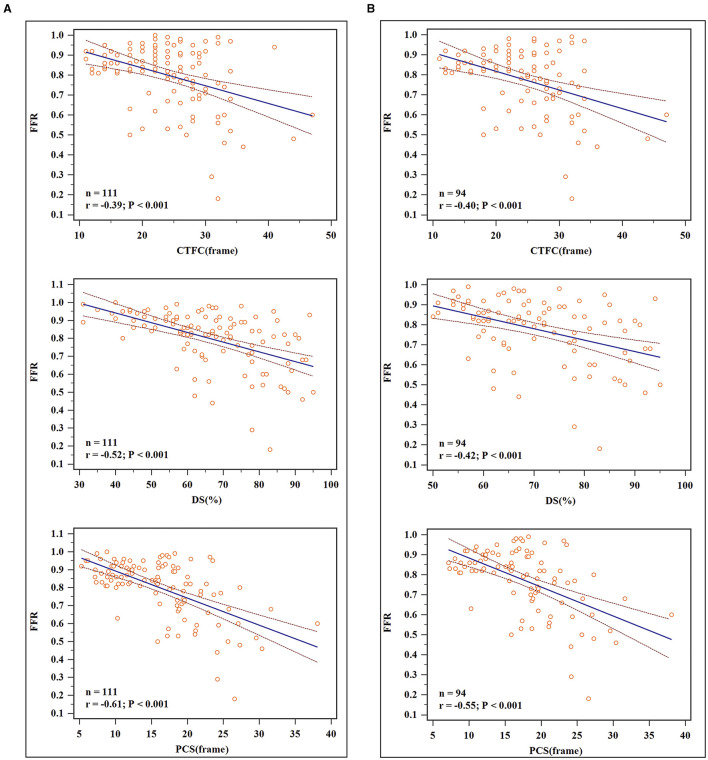
Scatterplots and linear regression results. **(A)** For all vessels: DS (*r* = −0.52), CTFC (*r* = −0.39) and PCS (*r* = −0.61) were correlated negatively with FFR. **(B)** For vessels with DS ≥ 50%: DS (*r* = −0.42), CTFC (*r* = 0.40), and PCS (*r* = −0.55) correlated weakly yet statistically significant with FFR.

**Figure 3:**
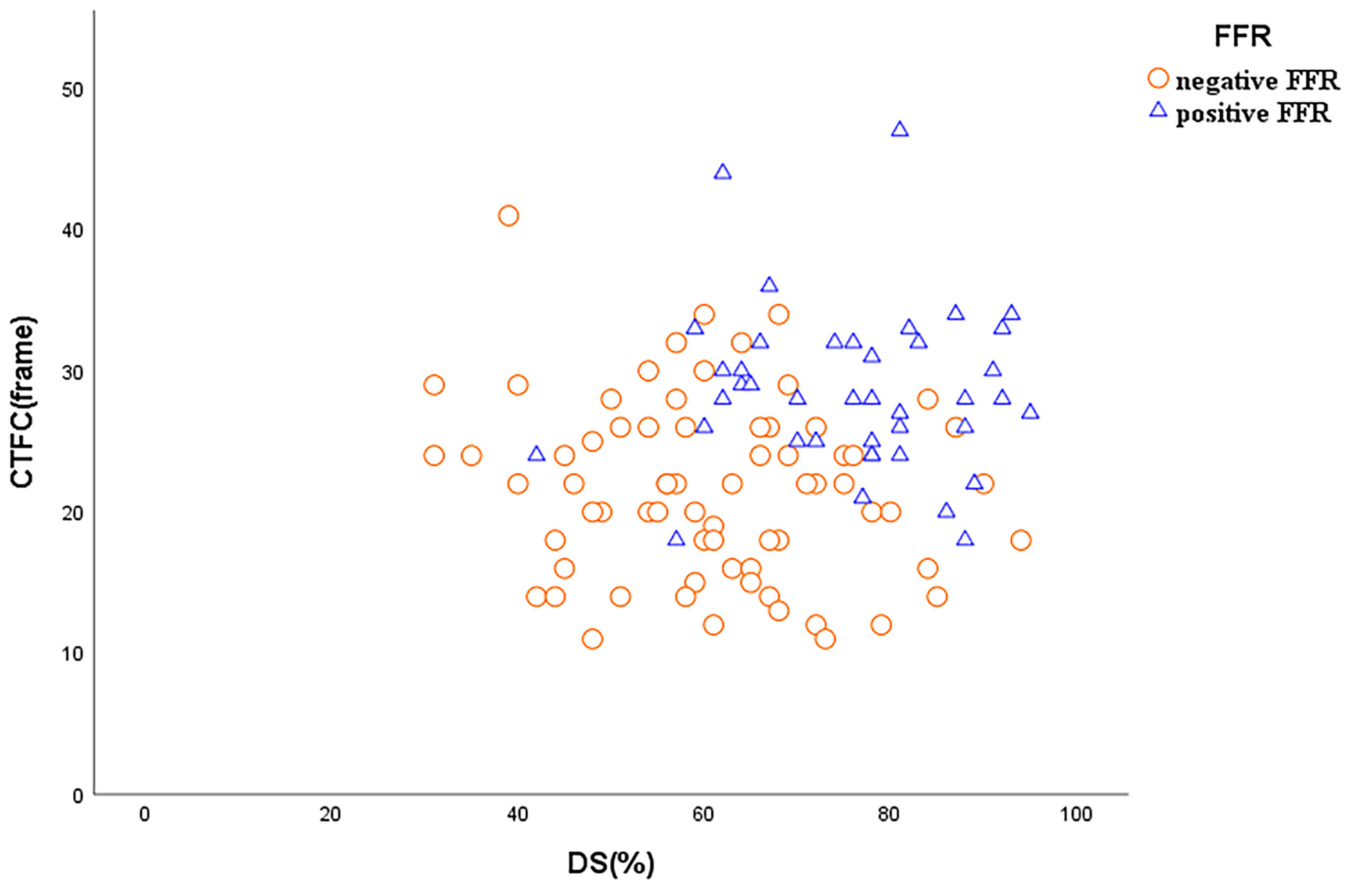
Scatterplots of DS and CTFC.

For all vessels, DS (*r* = −0.52), CTFC (*r* = −0.39) and PCS (*r* = −0.61) were correlated negatively with FFR (*P* < 0.05 for all). For vessels with stenosis ≥50%, DS (*r* = −0.42), CTFC (*r* = 0.40), and PCS (*r* = −0.55) correlated weakly yet statistically significant with FFR (Figure 2). As shown in Table 2, vessels with positive FFR exhibited significantly higher CTFC (28.56 vs. 21.64, *p* < 0.001) and PCS (21.67 vs. 13.04, *p* < 0.001) values than those with negative FFR. It was of note, the difference of CTFC values among the 3 stenotic levels were minor (Table 3). This phenomenon could be also illustrated from Figure 3.

**Table 2:** Angiographic characteristics in vessels according to invasive FFR.

**Variables**	**Negative FFR**	**Positive FFR**	***P-*value**
Vessels	70 (63)	41 (37)	
Stenosis severity			<0.001
Mild stenosis	16	1	
Intermediate stenosis	37	13	
Severe stenosis	17	27	
Lesion segment			0.213
Proximal	24	17	
Mid lesion	30	20	
Distal lesion	16	4	
**Lesion classifications**			
Simple lesions	35	15	0.170
Bifurcation lesions	13	12	0.193
Long lesions	29	16	0.803
DS (%)	60.94 ± 14.10	75.93 ± 11.98	<0.001
CTFC (frame)	21.64 ± 6.32	28.56 ± 5.82	<0.001
PCS (frame)	13.04 ± 4.48	21.67 ± 5.47	<0.001

Data are presented as the mean ± SD, or median (interquartile range). CTFC, the corrected TIMI frame count; DS, percent diameter stenosis; PCS, CTFC*DS FFR, Fractional flow reserve.

**Table 3:** Angiographic patterns in vessels according to ICA.

**Variables**	**Mild stenosis**	**Intermediate**	**Severe**	***P*-value**
		**stenosis**	**stenosis**	
CTFC (frame)	22.18 ± 7.04	24.22 ± 7.07	24.95 ± 6.84	0.381
PCS (frame)	9.18 ± 2.62	14.91 ± 4.68	20.45 ± 6.13	<0.001
FFR	0.93 ± 0.05	0.83 ± 0.13	0.72 ± 0.18	<0.001

Data are presented as the mean ± SD, CTFC, the corrected TIMI frame count; DS, percent diameter stenosis; FFR, Fractional flow reserve; PCS, CTFC*DS.

### Diagnostic Value for Flow-Limiting Coronary Stenosis

**Figure 4:**
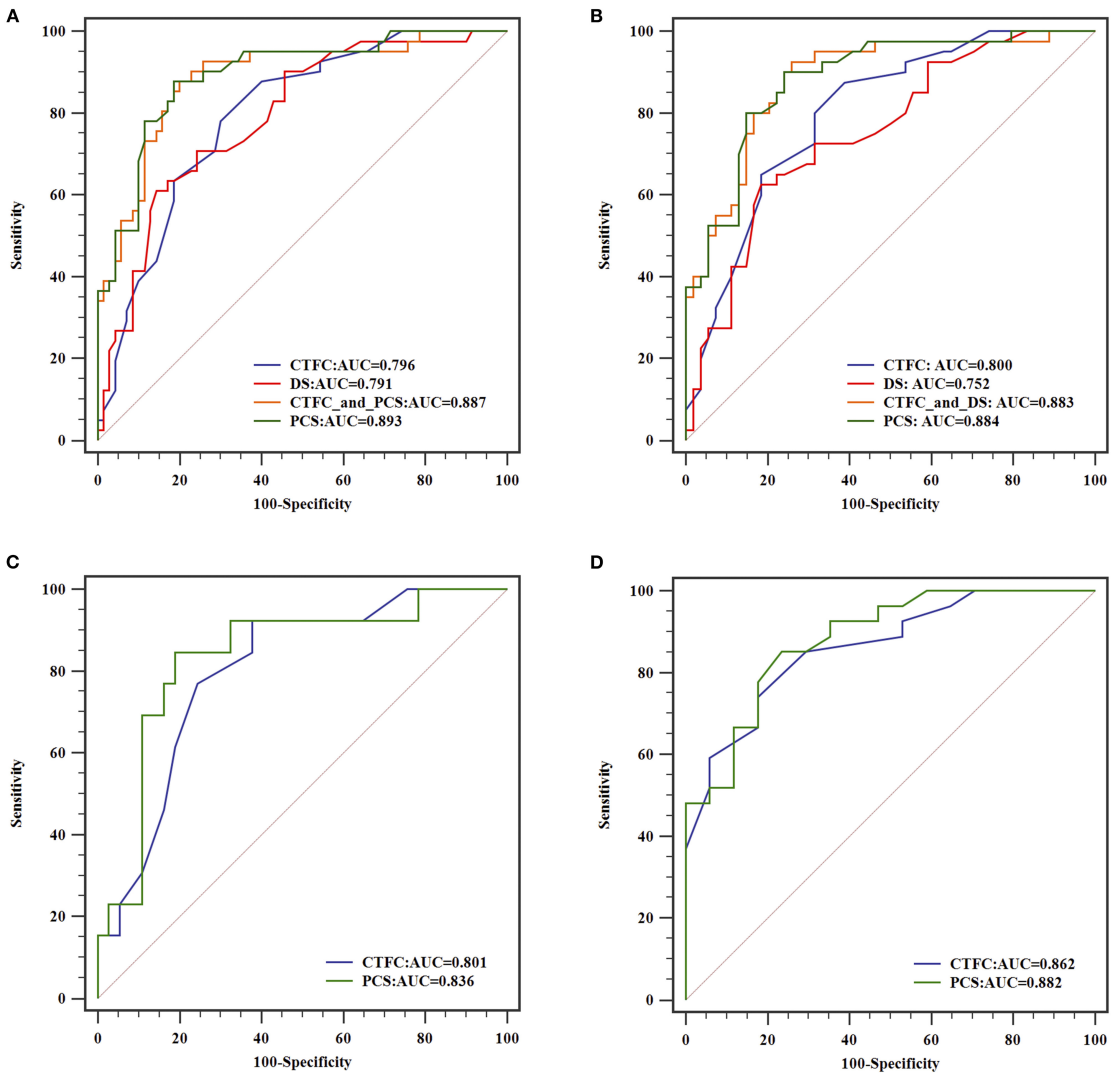
ROC curves of different models for diagnosing flow-limiting stenosis. From **(A–D)**, analysis of all coronary arteries, arteries with DS ≥ 50% stenosis, arteries with intermediate and severe stenosis were illustrated, respectively.

To analyze the power to diagnose flow-limiting coronary artery lesions, angiographic parameters that differed with statistical significance between positive and negative FFR groups were introduced into the binary logistic regression. After validation with forward LR selection, only CTFC and DS remained significant statistically. Then we select CTFC as the variable to build Model 1, and DS to build Model 2. CTFC and DS, the two variables, together formed Model 3. The new index PCS alone constructed Model 4. Receiver operating characteristic (ROC) analyses for these variables were shown in Figure 4. As shown in Table 4, for all vessels, CTFC and stenosis percentage could diagnose stenosis with positive FFR. Compared with Models 1 and 2, Model 3 increased the AUC, sensitivity, Youden's index, negative predictive value (NPV) (Table 5). The probability formula for differentiating stenosis with positive FFR was logit (p) = −13.040 + 0.222X1+ 0.100X2 (X1, CTFC; X2, DS). Compared with Model 3, PCS could raise the positive predictive value (PPV) from 57.1 to 72.0% and the DA from 51.4 to 82.9% despite the similar AUC, Youden's index, and NPV when the cutoff value was 17.16 (Table 5).

**Table 4:** The results of binary logistic regression in coronary arteries with positive and negative FFR.

	**Model 1**		**Model 2**		**Model 3**		**Model 4**	
	**OR (95% CI)**	***p***	**OR (95% CI)**	***p***	**OR (95% CI)**	***p***	**OR (95% CI)**	***P***
**Total vessels (*****n*** **=** **111)**								
CTFC	1.210 (1.112, 1.318)	<0.001			1.248 (1.126, 1.383)	<0.001		
DS			1.089 (1.049, 1.129)	<0.001	1.105 (1.055, 1.158)	<0.001		
PCS							1.443 (1.255, 1.659)	<0.001
**DS** **≥** **50% (*****n*** **=** **94)**								
CTFC	1.235 (1.122, 1.360)	<0.001			1.288 (1.141, 1.454)	<0.001		
Stenosis			1.089 (1.043, 1.136)	<0.001	1.114 (1.053, 1.179)	<0.001		
PCS							1.473 (1.254, 1.732)	<0.001
**50%** **≤** **DS** **≤** **70% (*****n*** **=** **50)**								
CTFC	1.225 (1.065, 1.409)	0.004			1.219 (1.059, 1.404)	0.006		
DS			1.130 (0.985, 1.296)	0.082	1.128 (0.967, 1.316)	0.125		
PCS							1.405 (1.131, 1.744)	0.002
**70%** **<** **DS** **≤** **95% (*****n*** **=** **44)**								
CTFC	1.395 (1.131, 1.719)	0.002			1.416 (1.130, 1.775)	0.003		
DS			1.094 (0.991, 1.209)	0.076	1.097 (0.965, 1.248)	0.158		
PCS							1.515 (1.165, 1.970)	0.002

CTFC, the corrected TIMI frame count; DS, percent diameter stenosis; FFR, Fractional flow reserve; PCS, CTFC*DS. CTFC, stenosis percentage and PCS separately constituted models 1, 2, and 4. CTFC and stenosis percentage were introduced at the same time to establish model 3.

**Table 5:** The results of ROC analysis in coronary arteries with positive and negative FFR.

**Model**	**AUC**	**Cut off**	**Sensitivity (%)**	**Specificity (%)**	**Youden's index**	**PPV (%)**	**NPV (%)**	**DA (%)**
**Total vessels (*****n*** **=** **111)**								
Model 1	0.796	24	78.0	70.0	0.480	56.3	89.4	70.3
Model 2	0.791	75	61.0	85.7	0.467	67.6	78.4	74.8
Model 3	0.887	0.365	87.8	80.0	0.678	57.1	90.0	51.4
Model 4	0.893	17.16	87.8	81.4	0.692	72.0	91.8	82.9
**Vessels with DS** **≥** **50% (*****n*** **=** **94)**								
Model 1	0.800	22	87.5	61.1	0.486	55.4	86.2	64.8
Model 2	0.752	75	62.5	81.5	0.440	68.4	75.0	72.3
Model 3	0.883	0.333	92.5	74.1	0.666	48.8	92.9	65
Model 4	0.884	17.16	90.0	85.9	0.759	72.9	89.1	80.9
**50%** **≤** **DS** **≤** **70% (*****n*** **=** **50)**								
Model 1	0.801	24	92.3	62.2	0.545	42.9	95.5	66.0
Model 4	0.836	17.16	84.6	81.1	0.657	61.1	93.8	82.0
**70%** **<** **DS** **≤** **95% (*****n*** **=** **44)**								
Model 1	0.862	24	74.1	82.4	0.564	82.1	75.0	79.5
Model 4	0.882	18.24	85.2	76.5	0.617	82.8	80.0	81.8

*DS, percent diameter stenosis; ROC, receiver operating characteristic curve; AUC, Area Under Curve; PPV, positive predictive value; NPV, negative predictive value; DA, diagnostic accuracy*.

For vessels with stenosis percentage ≥50%, CTFC had a higher sensitivity (87.5%), NPV (86.2%) and AUC (0.800) than DS, yet the specificity (61.1%), DA (64.8) and PPV (55.4%) was limited. PCS manifested higher additive value beyond Model 3 in specificity (85.9%), PPV (72.9%) and DA (80.9%) when cut-off value was 17.16.

When interrogating the vessels with intermediate stenosis, DS could not remain significant in Models 2 and 3. CTFC showed a higher sensitivity (92.3%), yet the specificity (62.2%) and DA (66.0%) were limited. PCS could differentiate 82.0% of cases at the cutoff value of 17.16 with higher specificity (81.1%) and AUC (0.836).

For vessels with severe stenosis, the value of CTFC was even dominant. At the cutoff value of 24, the specificity and PPV of CTFC were 82.4 and 82.1%, respectively. PCS was superior to CTFC in AUC (0.882), sensitivity (85.2%), Youden's index (0.617), and NPV (80.0%) when the cutoff value was 18.24.

### Intra- and Inter-observer Variabilities

**Figure 5:**
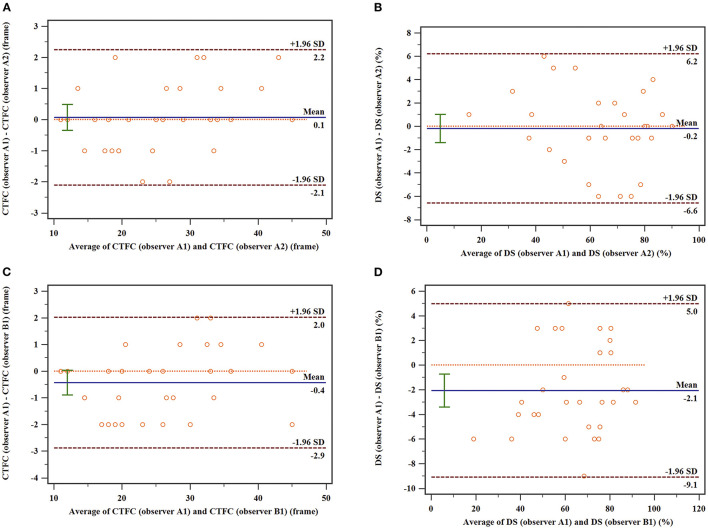
Bland–Altman plots of the intra-observer and inter-observer variability of CTFC. **(A)** Intraobserver variability in CTFC; **(B)** Intraobserver variability in DS; **(C)** interobserver variability in CTFC. **(D)** Interobserver variability in DS. The upper and lower dotted lines indicate the mean difference ± 1.96 SD, and the middle dotted line is used as the zero line, while the solid line depicts the mean difference between the two measurements.

The interclass and intraclass correlations coefficients for both CTFC and DS were excellent. For the intra-observer variability, the interclass correlations coefficients (ICC) of CTFC, and DS were 0.993, and 0.985, respectively. For the inter-observer variability, the ICC of CTFC and DS were 0.990, and 0.973, respectively. Intra- and inter-observer coefficient of variance (COV) for CTFC were 4.3 and 4.8%, respectively. Intra- and inter-observer COV for DS were 5.2 and 5.6%, respectively. The Bland-Altman plots were displayed in Figure 5.

## Discussion

The main finding of this study is that PCS improves the diagnostic accuracy of flow-limiting coronary stenosis by combining CTFC and stenosis information. Particularly, as the stenosis severity increased from intermediate to severe levels, the value of the stenosis percentage decreased significantly, whereas CTFC and PCS became more effective.

Stenosis percentage alone is recognized as not accurate enough to predict myocardial ischemia (3, 24). Our study indicated that only 26.0% of vessels with intermediate stenosis had functional significance. Even in coronary arteries with severe stenosis, 38.6% of lesions did not cause coronary blood flow limitation. These results were similar to the findings from previous studies (19, 25). In addition, 20–50% of patients with chest pain could have normal angiographic findings (26, 27). Studies have claimed the discordance between stenosis percentage and FFR, which may be due to several factors. First, the stenosis percentage only provides local site information, but the FFR reflects the entire epicardial resistance between the guide and pressure sensor. The FFR may be affected by other factors, such as different lesion shapes, lesion lengths, surface roughness, plaque eccentricity, and plaque ruptures of complex shapes (19). Second, compared with mild stenosis, intermediate or severe stenosis is more likely to have compensatory enlargement and collateral circulation. The vital reason for the disconnect between anatomy and physiology is related to the myocardial mass, which depends on the stenosis and vasodilatory capacity of the vascular bed (2). The reference diameter of stenosis accounts for the myocardial mass to some extent. For example, stenosis at the LAD is more likely to lead to hemodynamically significant lesions, even mild stenosis (2). In addition, stenosis at the local site does not reflect the vasodilatory ability of the microvasculature in the downstream area. Stated another way, the functional assessment of the coronary artery should no longer be limited to intermediate angiographic stenosis but should be considered in mild or severe stenosis assessed by coronary artery angiography.

Although FFR is recommended by the guidelines for functional assessment of epicardial coronary artery stenosis, the overall utilization of FFR is low (28, 29) due to the comparative complexity of the operation, risk of complications related to the wire maneuver, high cost, and poor tolerance to stress agents in some patients. The quantitative flow ratio (QFR) is a new method based on ICA that can calculate fractional flow reserve without using pressure wires or adenosine at the cost of an additional workstation with excellent computing power (23, 30, 31). However, QFR hasn't been widely used at present. Therefore, conventional coronary angiography is still the dominant guiding method of percutaneous coronary intervention (PCI) in vast catheter labs (32), which draws great attention to a more economical and practicable method to evaluate coronary artery functional stenosis.

CTFC is a simple, cost-effective, reproducible, and accurate method that has been proven to be valuable in evaluating the outcomes of percutaneous coronary interventions. The CTFC/minimal luminal diameter (MLD) ratio was reported by Stankovic to predict clinical and angiographic restenosis after percutaneous transluminal coronary angioplasty (PTCA) (12). Lower CTFC of the infarct-related artery immediately after revascularization was associated with better functional recovery of the infarcted myocardium and clinical outcome (11, 14). CTFC could provide independent prediction of hospitalization for angina in patients without obstructive CAD (33).

In essence, CTFC measures the velocity derived from frame count and contains specific location information for each vessel. Combined with the length of arteries measured with guidewires, CTFC could furnish flow assessments that are sensitive to slight changes in perfusion (34). There is a close linear relationship between CTFC and the distal mean peak velocity, flow volume, and coronary blood flow reserve (11, 12). CTFC is a critical factor in some emerging techniques for evaluating myocardial ischemia. In contrast-flow QFR calculations, TIMI frame count analysis was performed on the two diagnostic angiographs to obtain the hyperemic flow velocity (23). In other words, CTFC can be used as a simple quantitative indicator of coronary blood flow. Absolute coronary flow can be calculated by multiplying the average cross-sectional lumen area along the length of the artery to the TIMI landmark times the velocity (34). To simplify it, we introduced PCS to integrate structural and functional aspects of coronary arteries. For the culprit coronary artery, PCS can be regarded as the loss of coronary blood flow due to stenosis (Figure 1). Therefore, the increase in PCS of the coronary artery means a decrease in coronary blood flow. With FFR as the reference standard, at the same stenosis severity level, we found that it was the CTFC or PCS rather than the stenosis percentage that played a crucial role in diagnosing flow-limiting lesions. In particular, for lesions stenotic from 50 to 95%, the stenosis severity alone lost its accuracy to tell the blood flow shortage. Compared with CTFC or DS, PCS could not only improve the overall diagnostic accuracy but also raise the sensitivity, PPV, and NPV. PCS could integrate anatomical and physiological information effectively.

Our results suggested that PCS, as a comprehensive evaluation of CTFC and stenosis, may be an attractive option for functional severity of coronary artery stenosis assessment. In particular, if sufficient non-invasive assessments are not performed before ICA, this method can be used as an alternative method to evaluate the hemodynamic significance of the lesions.

## Limitations

To popularize and apply these findings, further large-scale prospective clinical trials are needed. As for serial or diffuse lesions, formula of PCS should be improved to identify the real flow-limiting “culprit lesion.” We did not evaluate short-term and long-term major cardiovascular events if the interventional strategy was changed based on PCS. CTFC might be interfered with by microcirculation disturbance. We did not study the relationship between CTFC, PCS and parameters reflecting micro-vessel disease, such as IMR, which is worth exploring in the future.

## Conclusions

As the PCS significantly improved the accuracy to diagnose flow-limiting coronary stenosis, it could be used as a simple and cost-effective tool for coronary artery blood flow functional evaluation.

## Data Availability Statement

The original contributions presented in the study are included in the article/supplementary material, further inquiries can be directed to the corresponding author/s.

## Ethics Statement

The studies involving human participants were reviewed and approved by the Ethics Committee of Scientific Research of Shandong University Qilu Hospital (KYLL-2016-336). The patients/participants provided their written informed consent to participate in this study.

## Author Contributions

XL, PZ, MZ, LL, JP, WY, and MD: implementation and data analysis. XL and PZ: drafting. PZ and MZ: conception, design, and data interpretation. PZ: revising it critically for important intellectual content and final approval of the manuscript submitted. All authors contributed to the article and approved the submitted version.

## Funding

This work was supported by the National Key Research and Development Program of China (2016YFC1300302) and Key Research and Development Program of Shandong Province (2020ZLYS05).

## Conflict of Interest

The authors declare that the research was conducted in the absence of any commercial or financial relationships that could be construed as a potential conflict of interest.

## Publisher's Note

All claims expressed in this article are solely those of the authors and do not necessarily represent those of their affiliated organizations, or those of the publisher, the editors and the reviewers. Any product that may be evaluated in this article, or claim that may be made by its manufacturer, is not guaranteed or endorsed by the publisher.
